# Identifying metabolic dysfunction-associated steatotic liver disease in patients with type 2 diabetes mellitus using clinic-based prediction tools

**DOI:** 10.3389/fmed.2024.1425145

**Published:** 2024-09-25

**Authors:** Juma Alkaabi, Bachar Afandi, Omar Alhaj, Darakhshan Kanwal, Adnan Agha

**Affiliations:** ^1^Internal Medicine Department, College of Medicine and Health Sciences, United Arab Emirates University, Al Ain, United Arab Emirates; ^2^Department of Endocrinology, Tawam Hospital, Al Ain, United Arab Emirates; ^3^Tawam Hospital, Al Ain, United Arab Emirates

**Keywords:** metabolic dysfunction-associated steatotic liver disease, diabetes mellitus, Hamaguchi criteria, NAFLD fibrosis score, fibrosis-4 index

## Abstract

**Background:**

Metabolic dysfunction-associated steatotic liver disease (MASLD), formerly known as non-alcoholic fatty liver disease (NAFLD), is a global cause of chronic liver disease. The prevalence of MASLD is high in patients with type 2 diabetes mellitus (T2DM). Various non-invasive tools such as the fibrosis-4 index (FIB-4) and NAFLD fibrosis score (NFS), liver ultrasound, and FibroScan can aid in the detection of liver fibrosis in MASLD, while the Hamaguchi ultrasound-based liver grading system has demonstrated high sensitivity and specificity comparable to liver biopsy.

**Objective:**

We assessed the frequency of MASLD in patients with T2DM using the liver ultrasound Hamaguchi score and the accuracy of NFS and Fib-4 in identifying MASLD.

**Patients and methods:**

We retrospectively collected data and reviewed the charts of all patients with T2DM who underwent liver ultrasound and laboratory tests during the past 5 years.

**Results:**

A total of 6,214 medical records were screened, and only 153 patients (68.6% women; mean age, 59 ± 12.2 years) fulfilled the selection criteria. MASLD was diagnosed using the Hamaguchi grading criteria in 45.1% of patients. A high/intermediate NFS had a higher sensitivity (79.7%) for diagnosing MASLD with a specificity of 10.7%, while a high/intermediate Fib-4 score showed only 30.4% sensitivity but a higher specificity of 54.8%.

**Conclusion:**

Our study indicates that MASLD is frequent in patients with T2DM, and clinical prediction tools such as NFS and Fib-4 can be applied in clinic/primary care settings with variable results.

## 1 Introduction

NAFLD is described as an infiltration of the liver by fat deposits, which is not related to commonly identifiable causes of hepatic steatosis (e.g., alcohol, viral, secondary to drugs, or autoimmune processes) and comprises a wide array of presentations ranging from fat accumulation (steatosis), fat accumulation with inflammation (steatohepatitis), also called non-alcoholic steatohepatitis (NASH), and architectural changes (fibrosis), which may lead to liver damage and shrinkage (cirrhosis); and is currently the commonest cause of chronic liver disease globally with a prevalence of 30% (1). Though most of the patients having NAFLD may not fully progress to NASH cirrhosis, the sheer volume of patients with NAFLD who still end up developing cirrhosis is still quite high and has now become the leading indication for patients undergoing liver transplantation in the West (2). The prevalence data for NAFLD worldwide suggest that some regions, such as the Middle-East region, have one of the highest rates of NAFLD being an indication for liver transplantation, up to 32%; however, there have been no large-scale specific studies conducted in the United Arab Emirates (3). T2DM is one of the most important risk factors associated with NAFLD, with a recent systematic review indicating the worldwide prevalence of NAFLD is 50–70% in patients having T2DM, with the highest prevalence seen in European countries, but again, there are no data available for our local Emirati population (4). In 2023, Delphi consensus redefined NAFLD and NASH and introduced an overarching term of steatotic liver disease that includes metabolic dysfunction-associated steatotic liver disease (MASLD), formerly known as NAFLD, metabolic dysfunction-associated steatohepatitis (MASH), formerly known as NASH, and metabolic and alcohol-related/associated liver disease (MetALD) to represent a separate group of patients with MASLD that consumes alcohol (140–350 g/week for women and 210–420 g/week for men) (5). MASLD was redefined and defined as hepatic steatosis identified by imaging or biopsy along with the presence of at least one cardiometabolic criteria, i.e., either overweight (raised body mass index (BMI) or increased waist circumference), glucose intolerance (T2DM or pre-diabetes or impaired fasting glucose), hypertension, low high-density lipoprotein, or raised triglycerides (5). Recent Delphi consensus on the nomenclature of steatotic liver disease suggested only the new terms, such as metabolic dysfunction-associated steatotic liver disease (MASLD) and MASH, be used to describe the current International Classification of Diseases Manual-10 codes of NAFLD and NASH, respectively (6). The gold standard for diagnosing MASLD and MASH is the histological evaluation after performing a liver biopsy (gold standard). However, this is an invasive procedure with a subsequent risk of complications (7). Other non-invasive methodologies for identifying MASLD include vibration-controlled transient-elastography (VCTE), which uses FibroScan to provide the measurement of the liver stiffness (LSM) expressed as kilopascals (kPa), and this can correlate accurately with advanced stages of liver fibrosis such as stage F3 (bridging fibrosis) and stage F4 (advanced scarring or cirrhosis), making it an alternative to the liver biopsy (8). Further non-invasive means to identify the MASLD include clinical/biochemical scoring systems such as FIB-4, NFS, enhanced liver fibrosis panel, Hepascore, alanine aminotransferase (ALT), aspartate aminotransferase (AST) to platelet ratio index (APRI), and imaging techniques such as VCTE, liver CT scan, and liver magnetic resonance elastography (MRE) (9). There are no current unanimously agreed-upon screening guidelines for MASLD in all patients with diabetes mellitus or risk factors. The American Association for the Study of Liver Disease recommends the Fib-4 score, whereas the European Association for the Study of Liver Disease recommends screening with blood tests, including ALT, NFS, and/or Fib-4, among others, for high-risk patients (10, 11).

Imaging modalities such as ultrasonography of the liver may be used for the evaluation of hepatic fat infiltration, and although it is a safe, low-cost, and non-invasive procedure that is most frequently utilized in clinical settings for other indications, its accuracy for liver fat quantification has not been established consistently (12). Hamaguchi et al. proposed an ultrasound liver grading system that scores based on hepatorenal echogenicity and liver brightness (score of 0–3), deep attenuation (score 0–2), and liver vessel blurring (score 0–1). This system enhances sensitivity and specificity for identifying MASLD (with liver steatosis >10%) to 97 and 100%, respectively, at a score ≥ 2, compared to liver biopsy (13). The Hamaguchi grading system also reduced the operator-dependent inter-observer variation with accuracy similar to liver computed tomography (13, 14).

NFS has been advocated as a non-invasive predictor of NAFLD fibrosis, which utilizes six clinical/biochemical variables, namely age, BMI, impaired fasting glucose or diabetes, platelet count, AST/ALT ratio, and albumin. NFS of 0.676 or more has been shown to have 67% sensitivity and 97% specificity to predict MASLD with an area under receiver operating curve (AUROC) of 0.85 in a recent large meta-analysis (15). A recent study compared various non-imaging predictive scores and imaging tools, such as MRE and VCTE, to liver histology for accurately diagnosing MASLD fibrosis. The results showed that NFS outperformed APRI, BARD, and the AST/ALT ratio, and was equally effective as Fib-4 and MRE in identifying liver fibrosis in patients with liver biopsy-proven MASLD (16). Current worldwide guidelines mostly recommend either Fib-4 or NFS as prediction tools for identifying patients with MASLD, but there is no consensus on which one is better (17).

Our study aimed to assess the frequency of metabolic dysfunction-associated steatotic disease (MASLD) in patients with T2DM in our local population using a liver ultrasound Hamaguchi score of ≥2 (which has comparable accuracy to liver biopsy), as well as to compare the performance of clinic-based non-invasive predictors, NFS and Fib-4, in identifying MASLD.

## 2 Patients and methods

This was a retrospective study design, approved by the local ethical committee (Approval Number. MF2058-2022-855), and it included all adult (aged 18 and above) patients of either gender, with T2DM, who had attended the diabetes clinic at Tawam Hospital from January 2017 to December 2021 and had undergone liver ultrasound and liver function tests (within 3 months of each other) during this period for any clinical indication other than MASLD. The patients with alcohol intake history (past or present), any evidence of existing hepatobiliary disease (biliary tract obstruction; hepatoma, liver cirrhosis secondary to infection or immune, or congenital), or having secondary diabetes (e.g., diabetes following pancreatitis) or type 1 diabetes mellitus were excluded. After reviewing over 6,214 medical records, 153 patients were identified, and their demographic details, clinical parameters, and biochemical test results, including liver function tests, albumin, bilirubin, glycated hemoglobin (HbA1c), prothrombin time, urea, and creatinine, were collected and recorded on a Microsoft Excel™ sheet. The ultrasound reports and images were reviewed for the visual estimation of fatty liver/MASLD, and these images were then reviewed by an additional expert ultrasound specialist radiologist. The Hamaguchi score for MASLD was calculated. A score of 2 or higher was considered diagnostic for the presence of MASLD. The Hamaguchi ultrasound liver grading system is based on the sum of points scored on three separate subsets (13). The first subset evaluates hepatorenal echogenicity and liver brightness: a score of 0 indicates the absence of bright liver and hepatorenal echo contrast, a score of 1 indicates the presence of either bright liver or hepatorenal echo contrast, a score of 2 indicates mild bright liver and positive hepatorenal echo contrast, and a score of 3 indicates severe bright liver and positive hepatorenal echo contrast. The second subset evaluates deep attenuation: a score of 0 is given for negative deep attenuation, a score of 1 for obscure diaphragm visualization, and a score of 2 for indistinguishable diaphragm. The third subset evaluates liver vessel blurring: a score of 0 is given for negative vessel blurring and a score of 1 is given when intrahepatic vessels are unclear and/or have a narrowed lumen.

The NFS was calculated based on the available formula from the website (http://gihep.com/calculators/hepatology/nafld-fibrosis-score/). Fib-4 and NFS were calculated, and the ability of these screening tests (low risk versus indeterminate or intermediate/high risk of significant fibrosis) to diagnose fatty liver disease was compared to that of the Hamaguchi scoring criteria. The cutoff values for Fib-4 and NFS as being abnormal were taken as ≥1.45 and ≥ −1.455, respectively. The sensitivity and specificity of Fib-4 and NFS for predicting a low risk of MASLD in patients with no fatty liver observed on ultrasound were also calculated. The data were analyzed using the SPSS package of Windows version 22. Pearson’s chi-square test was used to determine the effectiveness of non-invasive predictors, such as Fib-4 and NFS, in identifying MASLD based on ultrasound findings using the Hamaguchi criteria.

## 3 Results

A total of 6,214 patient medical records, including laboratory data and ultrasound imaging results, were reviewed; 5,926 patients were found to be ineligible due to incomplete biochemical tests (especially prothrombin time), and only 288 were found to be eligible as per our inclusion criteria. A total of 135 patients were excluded due to either the presence of type 1 or secondary diabetes and/or pre-existing liver disease (e.g., chronic active viral hepatitis) or a history of alcohol intake. For these selected 153 patients with available ultrasound images, a new expert radiologist reviewed all liver ultrasound scan images and applied the Hamaguchi criteria, identifying and confirming fatty liver disease in 69 patients (45.1%). Of these patients, 68.6% (105/153) were female, with a mean age of 59 ± SD 12.2 years. Additionally, 69.9% (107/153) were from the local Emirati population. The average duration of diabetes in all these patients was 12.2 ± SD 5.1 years, while 58.1% (89/153) had coexisting hypertension. Logistic regression was used to determine the correlation between quantitative variables, such as age and biochemical tests (platelet count, bilirubin, and HbA1c%) with the final outcome of MASLD. The chi-square test was used to assess the correlation of qualitative variables such as history of hypertension, gender, and the final outcome. No significant difference was observed. The details of the clinical and biochemical characteristics of patients with and without MASLD are shown in Table 1. A multi-logistic regression of all factors could not be performed due to the multicollinearity of factors such as the Hamaguchi score and various liver function tests, which are part of the NFS or Fib-4 scoring systems.

**Table 1:** Characteristics of the patients with and without advanced metabolic dysfunction-associated steatotic liver disease identified via Hamaguchi grading (score ≥ 2) (*n* = 153).

Characteristics	Patient with advanced metabolic dysfunction-associated fatty liver disease (*n* = 69)	Patients without advanced metabolic dysfunction-associated fatty liver disease (*n* = 84)
Age	57.5 + 13.1	60.7 ± 10.8
Gender	51 (73.9%) women	54 (64.3%) women
Ethnicity	46 (66.7%) Emirati	61 (72.6%)
Presence of hypertension	39 (56.5%)	50 (59.5%)
Duration of diabetes in years	12.1 ± 5.2	12.3 ± 5.1
BMI on the last clinic visit	31.4 ± 9.8	28.3 ± 6.6
Weight in kg	78.7 ± 20.5	72.5 ± 15.1
Microalbumin (mg/mmol)	184.7 ± 498.48	164.2 ± 368.7
Serum sodium mmol/L	138.6 ± 3.1	137.7 ± 4.3
Creatinine umol/L	109.0 ± 140.0	158.9 ± 182.7
Albumin g/dL	33.7 ± 5.2	31.9 ± 6.0
INR	1.1 ± 0.4	1.1 ± 0.3
Bilirubin μmol/L	7.9 ± 7.2	8.6 ± 10.0
ALT IU/L	30.1 ± 25.4	22.6 ± 21.5
AST IU/L	27.3 ± 17.1	24.1 ± 16.5
Platelets count × 10 ^9^ /L	270.6 ± 101.3	244.7 ± 92.5
Average of the last 3 HbA1c (in %)	7.8% ± 1.9%	7.9% ± 2.2
Last HbA1c prior to ultrasound	7.7% ± 2.1	7.8% ± 2.5
Hamaguchi grading score	3.19 ± 1.20	0.53 ± 0.5

Meanwhile, the ultrasound revealed that 45.1% (69/153) of the patients had MASLD according to the Hamaguchi criteria. An intermediate/high NFS was 79.7% sensitive for identifying MASLD on ultrasound, but its specificity was only 10.7%. Meanwhile, an intermediate/high Fib-4 had low sensitivity (30.4%) but higher specificity (54.8%) for identifying MASLD. However, both NFS and Fib-4 failed to achieve statistical significance on Pearson’s chi-square test for predicting MASLD (Table 2). Serum sodium <134 mmol/L was also predictive of MASLD with a specificity of 79.8%, but its sensitivity was only 10.1%.

**Table 2:** Comparison of the non-alcoholic fatty liver disease fibrosis score (NFS) and fibrosis-4 index (FIB-4) for predicting advanced metabolic dysfunction-associated steatotic liver disease (diagnosed via Hamaguchi grading on liver ultrasound).

Clinical prediction tool	Fatty liver*	No Fatty liver*	Sensitivity/specificity	*p*-value^3^
NFS^1^ low risk	14	9	79.7% sensitive and 10.7% specific	0.09
NFS^1^ intermediate/high risk	55	75		
FIB-4^2^ low risk	55	75	30.4% sensitive and 54.8% specific	0.06
FIB-4^2^ indeterminate/high risk	69	84		

*Identified as per liver ultrasound via Hamaguchi grading. 1. NFS, Non-alcoholic fatty liver disease fibrosis score. 2. FIB-4 = Fibrosis 4 index. 3. Significance is calculated via Pearson’s chi-square with a *p*-value of < 0.05 as significant.

## 4 Discussion

The prevalence of MASLD has surged globally, becoming a major public health concern due to its association with increasing morbidity and mortality (1). In our study, we wanted to assess the predictive value of non-invasive clinic-based tools, namely NFS and Fib-4, in identifying MASLD among patients with T2DM within our local Emirati population. Additionally, we compared these tools with the Hamaguchi ultrasound scoring system, which is known for its accuracy in identifying MASLD relative to liver biopsy (13, 14).

Our study deliberately concentrated on the utilization of cost-effective and readily available clinical tools, excluding more advanced imaging modalities such as MRI and FibroScan. While advanced techniques, such as MRI and FibroScan, are valuable in assessing liver fibrosis and steatosis, their availability and affordability can be limiting factors, particularly in resource-constrained healthcare settings (18, 19). By employing tools such as ultrasound and clinically derived scores like NFS and Fib-4, we aimed to align our study with the practical realities of clinical practice, especially in regions where access to high-end imaging technologies may be limited. The Hamaguchi ultrasound liver grading system, in particular, offers a feasible alternative with proven efficacy in identifying MASLD, boasting both sensitivity and specificity compared to more invasive procedures such as liver biopsy (14). By focusing on these clinically accessible tools, our study enhances the relevance of our findings to a broader spectrum of healthcare settings, facilitating the potential for wider implementation and impact on routine patient care.

Our findings underscore the substantial burden of MASLD in patients with T2DM, with 45.1% of patients identified as having MASLD according to the Hamaguchi criteria. This aligns with the global trends of increasingly emerging MASLD prevalence, as well as emphasizing the close association between T2DM and MASLD (4). The high frequency of MASLD in this cohort necessitates efficient yet economical screening tools to identify individuals at risk of advanced fibrosis or cirrhosis. Our study used and compared both NFS and Fib-4 as laboratory-based tools in the outpatient clinic setting, and it showed that NFS performed as a much more sensitive tool for predicting MASLD in our local population, exhibiting a sensitivity of 79.7%; however, its specificity was limited at 10.7%, suggesting a higher rate of false positives. On the other hand, the Fib-4 index demonstrated much lower sensitivity (30.4%) but higher specificity (54.8%). These results highlight the trade-off between sensitivity and specificity in non-invasive prediction tools for MASLD in T2DM patients. We wanted to see if having adding Fib-4 (indeterminate or high) score to NFS for screening MASLD could help improve the sensitivity/specificity of the prediction tool, but it only improved the sensitivity of NFS marginally from 79.7 to 81.7% with no change in specificity.

Comparing our results with the existing literature, NFS demonstrated favorable performance in identifying MASLD, which is something also seen in previous meta-analyses (20). However, the limitations of NFS, particularly its low specificity, should be considered in clinical practice. Although the Fib-4 index is currently recommended as a first-line screening test in the United States as part of the MASLD investigation pathway (21), our study found that it missed a substantial proportion of patients with MASLD. These observations emphasize the need for a multifaceted approach to MASLD screening, integrating various non-invasive tools and clinical parameters, including NFS, which can be used as a useful tool in primary care/outpatient setup to initially screen for MASLD.

The Hamaguchi ultrasound scoring system, used as a reference in our study, has proven valuable in identifying MASLD with high sensitivity and specificity (13, 14). The simplicity and non-invasive nature of this grading system make it an attractive option for routine clinical use. The feasibility and reliability of this system warrant further investigation, particularly in diverse populations. Furthermore, our study revealed a correlation between MASLD and serum sodium levels below 134 mmol/L; however, this did not achieve any statistical significance as the total number of patients having hyponatremia were low. This finding, however, introduces an intriguing avenue for further investigation.

### 4.1 Limitations

Despite contributing valuable insights, our study has limitations. First, the retrospective design may introduce selection bias, as patients with specific indications for liver-related concerns may have undergone liver ultrasound and laboratory evaluations. Second, the relatively small sample size from a single center limits the applicability/ generalisability of our findings to a broader Emirati population, and further large-scale prospective studies are required to confirm these results. The absence of liver biopsy data, which is the gold standard for MASLD diagnosis, poses a challenge in accurately characterizing the disease severity. Furthermore, the study only focused on NFS and Fib-4, and we could not calculate the fatty liver index, another clinical tool to predict steatosis, due to a lack of clinical data on waist circumference measurements. Although the current technological advances have allowed the use of newer imaging modalities such as MRI and FibroScan to accurately diagnose MASLD, as these are not easily accessible in primary care settings compared to ultrasound liver, we did not include them in our study. Furthermore, our study lacks longitudinal data, hindering the assessment of disease progression and the impact of interventions.

## 5 Conclusion

Our study describes the frequency of MASLD in patients with T2DM in our local population and evaluates the performance of non-invasive clinic-based prediction tools. MASLD was identified in approximately half of our patients with T2DM, which necessitates the need to use MASLD screening in clinical practice effectively. Using ultrasound with the Hamaguchi criteria offers an inexpensive and readily available tool, in most regional healthcare setups, for diagnosing MASLD and for referring to a specialist for further care and management. NFS and Fib-4 show distinct sensitivities and specificities, emphasizing the importance of considering these factors in clinical decision-making, especially in outpatient diabetes clinic settings/primary care. Our study indicated that NFS seemed to outperform Fib-4 in terms of its sensitivity in identifying patients with MASLD and thus could be used as a screening test in our population. However, future prospective studies with larger, diverse cohorts and longitudinal follow-up are crucial to validate these findings and develop evidence-based clinical guidelines for prospective clinic-based MASLD screening in patients with T2DM.

## Data Availability

The raw data supporting the conclusions of this article will be made available by the authors, without undue reservation.
